# Insight, symptom severity and medication adherence in bipolar disorder

**DOI:** 10.1192/j.eurpsy.2023.1448

**Published:** 2023-07-19

**Authors:** A. Abdelhamid, M. Nokrou, S. Bounouh, S. Belbachir, A. Ouanass

**Affiliations:** Psychiatry, Psychatric hospital Arrazi of Salé, Rabat, Morocco

## Abstract

**Introduction:**

Bipolar disorder is a mood disorder that requires adequate treatment, both acutely and for long-term prevention to avoid recurrence. A growing number of safe and effective medications, particularly thymoregulatory drugs and antipsychotics, can be used for the preventive and long-term treatment of bipolar disorder.

Insight, or awareness of the illness, has been little studied in bipolar disorder. It would seem that the presence of psychotic symptoms and manic polarity are linked to an alteration in clinical insight. The lack of insight could be linked to poor or non-adherence to treatment in psychiatric pathology including bipolar disorder.

**Objectives:**

Our aim is to study the correlation between the level of insight, symptom severity, and medication adherence in patients with bipolar disorder and compare them to those in the literature in order to allow a better therapeutic compliance for patients.

**Methods:**

It is a prospective descriptive study of a series of cases, carried out at the psychiatric hospital Ar-razi in Salé over a period of 4 months.

Our inclusion criteria are patients diagnosed with bipolar disorder according to DSM 5 criteria, aged over 18 years.

The data are collected during the psychiatric interview with the patient. Insight is assessed by the Beck Cognitive Insight Scale and the severity of the symptoms is assessed by the Clinical Global Impression.

**Results:**

Ongoing

**Conclusions:**

Ongoing

**Disclosure of Interest:**

None Declared

